# Experience of safety monitoring in the context of a prospective observational study of artemether-lumefantrine in rural Tanzania: lessons learned for pharmacovigilance reporting

**DOI:** 10.1186/1475-2875-9-205

**Published:** 2010-07-14

**Authors:** Abdunoor M Kabanywanyi, Nathan Mulure, Christopher Migoha, Aggrey Malila, Christian Lengeler, Raymond Schlienger, Blaise Genton

**Affiliations:** 1 Ifakara Health Institute, P.O. Box 78373, Kiko Avenue, Old Bagamoyo Road, Mikocheni, Dar es Salaam, Tanzania; 2 Novartis Pharma Inc, Nairobi, Kenya; 3 Tanzanian Food and Drugs Authority, Dar es Salaam, Tanzania; 4 Novartis Pharma AG, Basel, Switzerland; 5 Swiss Tropical and Public Health Institute, Basel, Switzerland

## Abstract

**Objectives:**

To identify and implement strategies that help meet safety monitoring requirements in the context of an observational study for artemether-lumefantrine (AL) administered as first-line treatment for uncomplicated malaria in rural Tanzania.

**Methods:**

Pharmacovigilance procedures were developed through collaboration between the investigating bodies, the relevant regulatory authority and the manufacturer of AL. Training and refresher sessions on the pharmacovigilance system were provided for healthcare workers from local health facilities and field recorders of the Ifakara Health Demographic Surveillance System (IHDSS). Three distinct channels for identification of adverse events (AEs) and serious adverse events (SAEs) were identified and implemented. Passive reporting took place through IHDSS and health care facilities, starting in October 2007. The third channel was through solicited reporting that was included in the context of a survey on AL as part of the ALIVE (**A**rtemether-**L**umefantrine **I**n **V**ulnerable patients: **E**xploring health impact) study (conducted only in March-April 2008).

**Results:**

Training was provided for 40 healthcare providers (with refresher training 18 months later) and for six field recorders. During the period 1^st ^September 2007 to 31^st ^March 2010, 67 AEs were reported including 52 under AL, five under sulphadoxine-pyrimethamine, one under metakelfin, two after antibiotics; the remaining seven were due to anti-pyretic or anti-parasite medications. Twenty patients experienced SAEs; in 16 cases, a relation to AL was suspected. Six of the 20 cases were reported within 24 hours of occurrence.

**Discussion:**

Safety monitoring and reporting is possible even in settings with weak health infrastructure. Reporting can be enhanced by regular and appropriate training of healthcare providers. SMS text alerts provide a practical solution to communication challenges.

**Conclusion:**

Experience gained in this setting could help to improve spontaneous reporting of AEs and SAEs to health authorities or marketing authorization holders.

## Background

Spontaneous reporting of suspected adverse drug reactions (ADRs) utilizing post-marketing surveillance or pharmacovigilance techniques during drug therapy is less applicable in many sub-Saharan African countries [1]. Pharmacovigilance is the science and activities relating to the detection, assessment, understanding and prevention of adverse effects or any other drug-related problem [2]. Pharmacovigilance plays a key role in ensuring that patients receive safe drugs. The knowledge of a drug's adverse effects can be increased by various means, including spontaneous reporting, intensive monitoring and database studies [3]. Post-marketing surveillance - which is often used synonymously for pharmacovigilance [4] and will be used throughout this article - is an important component of safety monitoring for drugs after they have been licensed for use. Detailed data on adverse events (AEs) are collected during the controlled clinical trials that are required for licensing, but however rigorous this process, the information collected cannot be regarded as entirely comprehensive due to the relatively restricted number of patients involved [1] and the exclusion criteria that are frequently applied, for example to omit pregnant women, young children, or elderly patients [5]. Accordingly, post-marketing surveillance, especially in the context of observational studies, can be a valuable source of additional safety data within a large patient population in a real-world setting.

The main factors limiting the implementation of pharmacovigilance in resource-limited settings include limited access to healthcare facilities, availability of most prescription drugs from the informal market, poor labeling of medications, high levels of illiteracy, poor record-keeping, a shortage of qualified healthcare professionals and a lack of awareness among healthcare workers of the need to identify and report suspected ADRs that occur during drug therapy. Post-marketing surveillance, including monitoring of anti-malarial drugs, is currently not undertaken in most sub-Saharan countries. A few countries in the region, including Tanzania, have managed to introduce a system of yellow cards, but this reporting process is still inefficient. Therefore, the benefits of pharmaco-epidemiological studies with planned, protocol-mandated collection of safety data may be particularly relevant in this region. However, careful attention must be paid to strategies that help to achieve effective safety monitoring during such studies.

Failure to properly assess the safety of a widely used drug such as a first-line anti-malarial treatment could result in public misperception and lead to problems with acceptability. This was seen in recent years with sulphadoxine-pyrimethamine (SP) [6]. Concerns about SP-related serious adverse skin reactions (e.g. Stevens-Johnson syndrome) led to unnecessary delays in the process of policy change in several African countries [7]. At the time, it was difficult for Ministries of Health to provide evidence-based information to the media and the public, and as a result public suspicion lingered for a long time.

Since then, following recommendations from the WHO that artemisinin-based combination therapy (ACT) be used as first-line treatment of uncomplicated malaria [8], artemether-lumefantrine (AL, Coartem^®^, Novartis Pharma AG, Basel, Switzerland) has been widely adopted throughout sub-Saharan Africa as first-line treatment for uncomplicated *Plasmodium falciparum *malaria. The efficacy and safety of AL have been extensively documented in clinical trials [9-14], but safety data for AL are currently limited when deployed on a large scale outside controlled clinical trials.

In November 2006, Tanzania adopted AL as first-line anti-malarial therapy as part of its national policy. Following this decision, the ALIVE (**A**rtemether-**L**umefantrine **I**n **V**ulnerable patients: **E**xploring health impact) study was initiated to evaluate the impact of implementing AL as first-line malaria treatment in a rural, malaria-endemic region of the country. ALIVE is an observational study undertaken by the Ifakara Health Institute (IHI) and the Swiss Tropical and Public Health Institute, and sponsored by Novartis Pharma AG and the Novartis Foundation for Sustainable Development. As for any study of this type, specific requirements for safety monitoring were specified in the protocol, but the challenges in meeting these requirements were recognized.

This paper describes how the pharmacovigilance requirements of the ALIVE study were being addressed through innovative initiatives that included dedicated training of relevant healthcare workers and community longitudinal demographic surveillances recorders. The use of short message service (SMS) text alerts was also encouraged. This may provide a potential model to ensure compliance with safety reporting requirements in other observational studies or more general post-marketing surveillance programmes.

## Methods

### The ALIVE study

ALIVE is a prospective, observational, community-based, longitudinal, demographic surveillance study in adults and children, undertaken to assess the impact of AL on malaria morbidity and mortality in a rural, malaria-endemic area of Tanzania when used as first-line treatment for uncomplicated malaria. Since first-line use of AL was adopted in Tanzania as national treatment policy in late 2006, it has been distributed to health facilities for use twice daily for three days to all patients ≥3 months of age with a clinical diagnosis of uncomplicated malaria. The first dose is administered under supervision at the health facility.

The primary objective of the study is to assess the effect of AL on all-cause mortality in infants and children aged ≥3 months (and >5 kg) and <5 years old compared to historical data using SP. Secondary objectives include the assessment of overall and malaria-related health facility attendance rate in children and in adults. This study also provided a framework for assessment of patient satisfaction, adherence to the AL regimen in both children and adults using a structured questionnaire, and safety monitoring of AL [15].

The study is taking place over a five-year period (2007-2011) in two rural districts of Tanzania (Ulanga and Kilombero). The ALIVE study population comprises the population of the Ifakara Health Demographic Surveillance System (IHDSS) in the Ulanga and Kilombero Districts, which numbered approximately 82,000 at the start of the study. The study area is characterized by monsoon tropical rains that fall from December to May, leading to an average annual rainfall of 1,200 mm. Malaria transmission ranges from intense to moderate and transmission is perennial, peaking after the period of long rains with little seasonal variation [16]. Across the study area there are 25 villages and 25 health facilities that include health posts, dispensaries, health centres and hospitals, with varying quality of care.

The ALIVE study is conducted in compliance with the Declaration of Helsinki following approval by the institutional review board of IHI and the Tanzanian National Institute for Medical Research (NIMR).

### Ifakara health demographic surveillance system

The IHI runs a well-established demographic surveillance system covering parts of the Ulanga and Kilombero districts in the ALIVE study, whereby standardized information on pregnancies, births, deaths and migrations are collected every four months by trained field recorders who visit each of the approximately 19,000 households in the surveillance areas of the two districts [17]. A complete household survey is performed annually to update the IHDSS database with socioeconomic and other key indicators. The IHDSS is being used to collect selected outcomes data in the context of the ALIVE study. The IHDSS unit is part of the Indepth-Network, a global network of 37 field sites in Africa and Asia focused on health and population research [18].

### Pharmacovigilance monitoring

In the context of this study, an AE is defined as '*unfavourable and unintended sign including an abnormal laboratory finding, symptom or disease associated with the use of a medical treatment or procedure, regardless of whether it is considered related to the medical treatment or procedure, that occurs during the course of the study'*. A serious adverse event (SAE) is defined as '*an undesirable sign, symptom or medical condition which is fatal or life-threatening, requires or prolongs hospitalization, results in persistent or significant disability/incapacity, constitutes a congenital abnormality or a birth defect, is medically significant, or may jeopardize the subject and may require medical or surgical intervention to prevent one of the outcomes listed previously'*. A suspected causality assessment is defined as follows: '*The temporal relationship of the clinical event to trial drug administration makes a causal relationship possible, and other drugs, therapeutic interventions or underlying conditions do not provide a sufficient explanation for the observed event'*.

**Figure 1:**
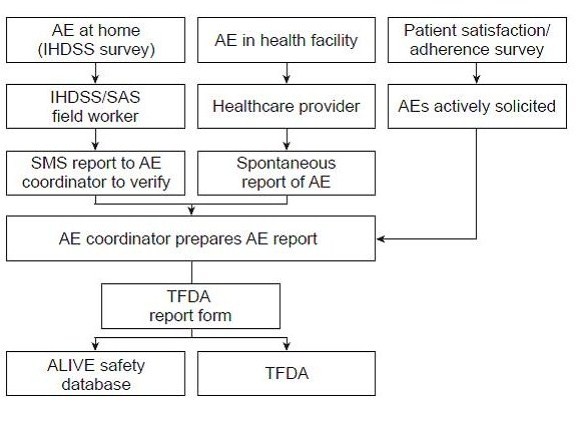
**Reporting channels for adverse events (AE)**. IHDSS, Ifakara Health Demographic Surveillance System; TFDA, Tanzanian Food and Drugs Authority; HH, Household; HF, Health Facility; SAS, Satisfaction/Adherence Survey.

The protocol included three channels through which AEs and/or SAEs can be identified and reported (Figure 1), to ensure that reporting is compliant with the regulations of the Tanzanian Food and Drugs Authority (TFDA) and with standard operating procedures of Novartis, the manufacturer of AL and the study sponsor. Of these, the first two channels (IHDSS and health facilities) employ passive pharmacovigilance while the third (patient satisfaction/adherence survey) actively solicits information on AEs and SAEs. Standard reporting forms from the TFDA were used to collect data on AEs. Standard Novartis SAE forms were, in addition, used to report SAEs.

#### Safety data from patients of all ages were reported

1. *IHDSS (Level I)*. Field reporters in the IHDSS (who are permanent residents in the area) have been instructed that for any AL-associated AE (regardless of seriousness) spontaneously brought to their attention, they should complete a TFDA form, and recommend to the reporter/patient that the event be reported to the health facility. The TFDA form is delivered to the local health facility, where additional details are added if possible. In the event of an SAE, a Novartis SAE form is additionally completed, and the SAE is assessed for causality by the physician and forwarded to Novartis. Additionally, in the event of an SAE being identified by IHDSS reports, IHDSS interviewers are trained to advise the patient to attend the local health facility immediately.

2. *Health facilities (Level II)*. Health professionals working in health facilities (i.e. medical officers, pharmacists, clinical/maternal and child health nurses, or laboratory staff) have also been instructed to complete a TFDA form for any spontaneously reported AL-associated AE, regardless of seriousness. In the event of an SAE, a Novartis SAE form is additionally completed. Assessment of causality by the treating physician is undertaken. If AL is administered during pregnancy, a specific Novartis pregnancy form is completed.

3. *Patient satisfaction/adherence survey (Level III)*. During the patient satisfaction/adherence survey that involved 552 malaria patients and was conducted between March-April 2008, information was actively solicited on AEs and SAEss, and recorded on the TFDA form (AEs) and the Novartis SAE form [15]. If an SAE is suspected after assessment by a treating physician, the form is submitted to Novartis.

The TFDA forms are collected monthly from health facilities and transmitted to the TFDA safety desk in Dar es Salaam by postal mailing.

**Figure 2:**
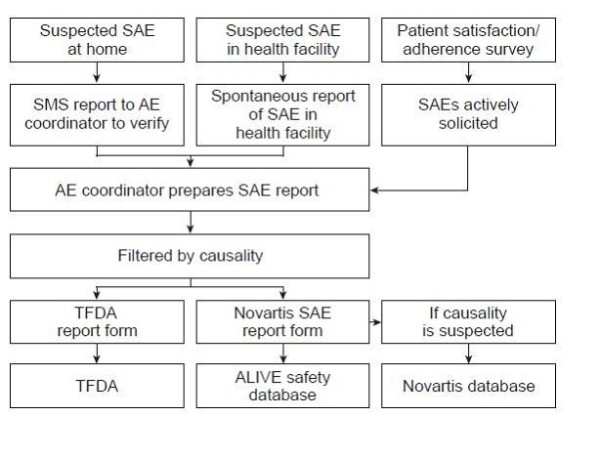
**Reporting channels for serious adverse events (SAE)**. IHDSS, Ifakara Health Demographic Surveillance System; TFDA, Tanzanian Food and Drugs Authority; HH, Household; HF, Health Facility; SAS, Satisfaction/Adherence Survey.

From Ifakara, all SAE forms are transmitted to the local East African Novartis safety office, by fax or email. All data are entered to a specific ALIVE safety database held at the IHI. The ALIVE safety coordinator also actively follows up the patients for verification and assessment and, if necessary, the safety coordinator refers the patient to a health facility for further assessment and management. In the event of incomplete information from the relevant healthcare providers, the ALIVE coordinator may visit the patient and complete all necessary forms on site. All TFDA and Novartis SAE forms are also forwarded to the TFDA (Figures 1 and 2).

The TFDA form requires standard reporting information (e.g. patient demographics, description of the event, suspected and concomitant drug(s), management and outcome of the event etc). The Novartis SAE form, which was field-tested by IHDSS and was found to be consistent with the TFDA report form, requires information on the diagnosis and description of the event, how the event met criteria for classification as a SAE, treatment doses at or before onset of the SAE, therapy dates, past medical history, relevant concomitant drugs, relevant laboratory values, investigator's causality assessment (i.e. suspected [possibly or probably related to study drug] or not suspected) and outcome. If a causality assessment is missing when an SAE is reported either at a health facility or during the patient satisfaction/adherence survey, the SAE form is sent to Novartis for provisional reporting.

### SMS reporting

In order to ensure timely reporting of AEs and SAEs, IHDSS field recorders or health facility staff were encouraged to send a mobile telephone SMS text alert to the ALIVE safety coordinator immediately when an AE or SAE is identified, prior to submitting the physical reporting form. The SMS includes a summary of patient demographics, date and type of event. Using this information, the safety coordinator then uses SMS texting to alert the local East African Novartis safety office and as necessary sends a fax or scanned copy of the initial report by email. Reminders to IHDSS field reporters and staff at health facilities that completed forms are required within the proscribed time frame (i.e. initial report within 24 hours of event and follow-up report not later than 15 days after the initial report) are also sent through SMS alerts.

### Training for healthcare workers

In October 2007, a one-day training session was provided for healthcare providers from health facilities located in the two districts in which the ALIVE study was undertaken. Participants were divided in to two groups, each trained separately once during each of the two consecutive training days. Each session comprised at least 35 persons, from both of the districts that host IHDSS (Kilombero and Ulanga). This training was conducted jointly by facilitators from the investigating bodies (IHI and the Swiss Tropical and Public Health Institute), the regulatory authority (TFDA) and the drug manufacturer (Novartis Pharma AG). Trainees were designated as focal persons for their health facilities after training. During the training session, delegates were instructed on how to identify AEs and SAEs, procedures for completing the relevant forms, and the reporting channels (Table 1). All reporting forms were presented and delegates were shown how to complete each one in a step-by-step process. Plenary presentations were followed by small-group breakout sessions in which case studies provided an insight into the identification and handling of AEs and SAEs and how to report them in a timely manner in compliance with the requirements of TFDA and Novartis. Delegates received training materials, handouts and copies of all relevant reporting forms.

**Table 1:** Content of training programme for healthcare workers at health facilities

Topic	Content
**Study drug (AL)**	Indications and dosage
	Contraindications
	Drug interactions
	Use in pregnancy/lactation
	Common ADRs (frequency > 10%)
	Special precautions
**Study objectives**	Primary and secondary objectives of the observational study (ALIVE)
**ADRs, AEs & SAEs**	Minimum reporting requirements
	Definitions of ADRs, AEs & SAEs (including congenital abnormalities & birth defects)
	Detection and recognition of ADRs, AEs & SAEs
**Reporting of AEs & SAEs**	Data collection requirements
	TFDA reporting form
	Novartis SAE reporting form

ADR = adverse drug reaction; AE = adverse event; AL = artemether-lumefantrine; ALIVE = **A**rtemether-**L**umefantrine **I**n **V**ulnerable patients: **E**xploring health impact; SAE = serious adverse event; TFDA = Tanzanian Food and Drugs Authority. ADRs were defined as 'all noxious and unintended responses to a medicinal product related to any dose. The responses to a medicinal product means that a causal relationship between a medicinal product and an adverse event is at least a reasonable possibility i.e. the relationship cannot be ruled out'

Eighteen months later (April 2009) another very similar training session was undertaken by experts from Novartis Pharma AG, Swiss Tropical and Public Health Institute, IHI and TFDA. This training included more details and emphasis on expedited reporting and causality assessments.

A similar package of training materials was also provided to IHDSS field recorders individually in the field by the ALIVE safety coordinator. In addition, a retraining session was provided for IHDSS field recorders, again undertaken by IHI with materials developed jointly by the Swiss Tropical and Public Health Institute, the TFDA and Novartis. The session covered the definition of safety terms (AEs, ADRs and SAEs), explained the reporting forms, and described the reporting procedures.

## Results

The first training session, in October 2007 was attended by 40 healthcare providers from health facilities in the study districts. The second training session was attended by 35 healthcare providers, of whom more than half had not attended the first training course because they had recently been relocated from facilities outside the ALIVE study area or returned from college. Six IHDSS field recorders attended the IHDSS training session and all IHDSS field recorders received reporting materials.

**Figure 3:**
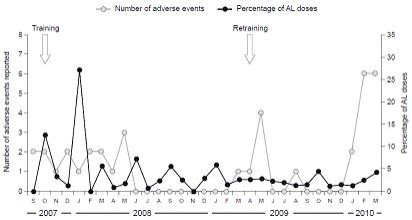
**Number of adverse events (AEs) reported per month during 1^st ^September 2007 to 31^st ^March 2010 indicated on the left-hand y-axis**. The right-hand y-axis indicates the percentage of AL tablets prescribed per month, with the total number of AL tablets during 1^st ^September 2007 to 31^st ^March 2010 as the denominator.

Pharmacovigilance activity from 1^st ^September 2007 to 31^st ^March 2010 is described in the following sections. Within this period, 67 AEs were reported across the total patient population, of which seven were identified through Level I (passive surveillance), 59 through Level II (passive surveillance) and the remainder through Level III (active surveillance). Among the 59 AEs that were reported through Level II, nearly 24% were exposure to AL during pregnancy. Eleven exposures occurred during the second trimester and the remaining three occurred in the third trimester. One of the three AEs that took place in the third trimester was a stillbirth, which occurred four weeks after AL exposure during the 34^th ^week of pregnancy with no suspected relationship with AL. The other exposures were not associated with any adverse pregnancy outcome. The reporting rate peaked shortly after the first training course, subsequently declining until after the second training course in April 2009 (Figure 3).

Of the 67 AEs reported, 52 occurred after AL therapy, five after SP, one after metakelfin, and another one after amodiaquine and paracetamol. Of the other AEs, one occurred after penicillin injection, two after paracetamol, four after ivermectin and one after administration of amoxicillin. During the same period (1^st ^September 2007 to 31^st ^March 2010), a total of 181,609 patients with suspected malaria reported to health facilities in the ALIVE study area and received AL as first-line treatment for malaria. The reporting rate of AL-associated AEs was therefore 28.6 per 100,000 AL-exposed patients. The AEs that were recorded after AL included vomiting (5 cases), itching and/or rash (21 cases); difficulty breathing, convulsion and headache occurred in 12 cases. The other 14 AEs recorded after AL administration, which occurred alone or in combination, were high fever (2), dyspnoea (2), fatigue (3), dizziness (2), paraplegia (1) and a swollen eyelid (1). Others were insomnia (1), stiffness of joints and neck (1) and dysuria (1). In all cases occurrences were reversible and regressed with malaria symptoms. The five AEs that occurred after SP were mild erythematic skin lesions that did not progress to Stevens-Johnson syndrome. The AEs seen following treatment with penicillin and amoxicillin were both rashes.

A total of 20 patients were reported who experienced SAEs during September 2007 to March 2010 (Table 2). In 16 cases, a relation to AL was suspected. All but two patients recovered; in the two cases where the patient died and the one in which a stillbirth occurred, the SAE was not classified as having a suspected relation to AL. Of the 20 patients with SAEs, 6 cases were notified to the ALIVE safety database within 24 hours of occurrence. Twelve of these cases were detected at the health facilities and seven at home through IHDSS surveys (passive surveillance). Only one case was detected at home during the patient satisfaction/adherence survey (active surveillance) by a research field interviewer and was thus reported directly to the safety coordinator in Ifakara. Two patients died. One was a 4-year-old child who died in hospital on the third day after admission. The patient was diagnosed with malaria at a secondary health care facility and given two tablets of AL, then referred to the tertiary health care facility where severe malaria was diagnosed on the day of referral, together with symptoms of convulsion and cough. AL was stopped and the patient put on quinine injection. Two days later, the patient died in the hospital, with the verbal autopsy giving the cause of death as difficulty in breathing. The second death occurred in an infant of five months, who was initially treated with AL and amoxicillin at a secondary health care facility one day before death. On the next day the infant's condition deteriorated and AL discontinued, and the infant was then treated at the nearby primary health care facility as a case of severe malaria and pneumonia using quinine and penicillin injection. The patient was not referred to hospital on time and as a result died at home due to severe respiratory distress.

**Table 2:** Patient characteristics, type, timing and outcomes of serious adverse events (SAEs) reported during 1^st ^September 2007 to 31^st ^March 2010.

Sex	Age (years)	**SAE**^**a**^	Interval between event and recording	Outcome	Level of reporting
**Female**	37	Severe headache & vomiting	1 days	Recovered	II
**Female**	12	Dyspnoea & vomiting	1 day	Recovered	II
**Male**	2	Twitching	1 days	Recovered	II
**Female**	4	Severe vomiting	4 days	Recovered	II
**Female**	2	Generalized itching/rash^b^	>60 days ^a^	Recovered	II
**Female**	1	Respiratory distress	3 days	Died; not classified as suspected in Novartis safety database	III
**Female**	4	Convulsion	9 days	Died; not classified as suspected in Novartis safety database	II
**Female**	46	Dyspnoea	1 day	Recovered	II
**Male**	38	Generalized rash^b^	7 days	Recovered	II
**Female**	47	Generalized rash^b^	1 day	Recovered	II
**Male**	13	Dyspnoea & swollen eyelids	1 day	Recovered	II
**Male**	7 months	Skin rashes	20 days	Recovered	I
**Female**	17	Paraplegia	>60 days	Recovered	I
**Female**	5	Joint stiffness	>60 days	Recovered	I
**Male**	10	Dizziness & headache	30 days	Recovered	I
**Female**	32	Skin rashes & amnesia	>60 days	Recovered	I
**Female**	11	Skin rashes	60 days	Recovered	I
**Male**	7	Skin rashes	60 days	Recovered	I
**Female**	47	Skin Rashes	10 days	Recovered	II
**Female**	34 weeks (gestational age)	Stillbirth	>60 days	Stillbirth; not classified as suspected in Novartis safety database	II

All events were recorded following AL treatment and were classified as SAEs by presenting with any or all of the following: (a) fatal or life-threatening (b) prolonging hospitalization (c) resulting in persistent or significant disability/incapacity (d) may jeopardize the subject or (e) may require medical or surgical intervention to prevent one of the previous outcomes.

^a ^Report was initially misplaced at the health facility and not made available to safety coordinator on time

^b ^Skin depigmentation for an extended period, which was considered to constitute significant incapacity Level of reporting: Level I, IHDSS; Level II, health facilities; Level III, patient satisfaction/adherence survey

## Discussion

Observational studies such as ALIVE offer the opportunity to obtain safety data on marketed drugs in settings that extend beyond the relatively small and selected populations that are assessed in randomized, controlled clinical trials. Undertaking a prospective observational study, however, requires careful consideration of how to meet the protocol-specified safety monitoring requirements of the relevant health authorities and the manufacturer. Particular challenges are faced in establishing pharmacovigilance monitoring in many rural areas of sub-Saharan Africa, where existing health services and patient access are often limited and often coupled with low awareness and motivation of healthcare staff about the need to report potential safety problems that occur during drug treatment.

The current paper describes how a training programme for healthcare personnel, accompanied by provision of training and reporting materials and the use of SMS text alerts, was adopted to support the safety monitoring for a large-scale prospective observational study of the use of AL for uncomplicated *P. falciparum *malaria in a rural area of Tanzania. The benefits of training were demonstrated by the increased AE reporting rate observed after both initial and follow-up training sessions, and by the appropriate nature of the events reported and adequacy of data provided. The decline in reporting during the 18-month interval between the initial and follow-up training highlights the critical nature of repeated training and reminders. The upsurge in AE reporting and subsequent downturn eight months later is a significant cause for concern, especially for a programme that has to be incorporated into the routine health service delivery system. In particular, the type of training session conducted in this study may not be sustainable within the standard health service given the inherent costs and time burden for the already overstretched system. In the ALIVE study, however, this approach was adopted because there was no efficient and cheaper comparable alternative. The view of the authors is that pharmacovigilance reporting must be included in the curricula of medical schools and be part of the job description of health care workers. Where possible, it should be included in the health information management system in the set up of developing countries to maximize capture of AEs. This work provides a solid basis for a recently planned project to establish pharmacovigilance reporting in eight sites in Burkina Faso, Ghana, Mozambique and Tanzania: the INDEPTH Phase IV Safety and Effectiveness Studies Platform (INESS) [19].

For each AE reported, minimum reporting requirements were met, i.e. an identifiable reporter, an identifiable patient, a suspected product, and an AE. SAEs were reported in 20 patients, of whom five were given intravenous quinine after referral to tertiary health facilities and subsequently recovered, suggesting a missed diagnosis of complicated malaria for which AL was not the appropriate treatment [20]. Rash, as reported here in 21 cases, is a recognized and frequent AE associated with artemesinin-based combination therapy [9,21-23]. There were 14 cases of AL treatment reported in pregnant women during the period described. For the single case of stillbirth that occurred during AL exposure, medical records were sparse which made the cause of stillbirth difficult to establish. The mother may have been HIV-positive. In addition, she was given quinine at week 32 of pregnancy, followed four days later by vaginal bleeding and then stillbirth, suggesting that quinine may have played a causative role.

A markedly lower reporting rate is routinely observed in observational post-marketing studies that use passive pharmacovigilance monitoring (whereby AEs are only reported spontaneously by patients or carers to health professionals) compared to clinical trials in which data on AEs are collected actively, even in countries with well-established spontaneous reporting systems [5,24]. In some sub-Saharan African countries, the challenge of capturing safety data may be even more profound: in a recent observational study of first-line AL use in rural Ethiopia, not a single AE was reported spontaneously over a two-year period despite over 200,000 individuals presenting with suspected malaria [25]. Reporting rates of AEs in the ALIVE study - which mainly relied on passive pharmacovigilance - were higher than in the Ethiopian study; however, they were still low taking into account that over 180,000 AL treatments were administered during the observation period. This may be partly explained by the good overall safety profile of AL, with most of the AEs observed in clinical trials being related to malaria itself rather than to AL exposure [21,26]. Most of the AEs occurring after AL exposure in the current study are similar in nature to those that were observed in the pre-registration clinical trials of the drug [21,27].

Most of the reports of AEs made under the ALIVE pharmacovigilance programme reported in this paper were captured through the passive surveillance route, but it should be borne in mind that passive pharmacovigilance systems have various limitations. Passive pharmacovigilance can be particularly challenging in poorly educated, remote communities some distance from the nearest health facility and with a low number of trained healthcare workers. These factors are likely to have been an important cause contributing to low reporting rates in this study as well. Other passive monitoring initiatives, such as the promotion of 'yellow cards' in a rural area of Mozambique [28] and a malaria pharmacovigilance programme in South Africa [29] have also shown low reporting rates (of ADRs), underscoring the challenge of effective safety data collection during anti-malarial therapy in Africa outside the context of clinical trials.

The use of active surveillance, with prospective follow-up of the treated population, is ideal but unrealistic on a large scale due to cost and manpower requirements. In this study, however, active surveillance was applied only during a survey that investigated the feasibility and acceptability of AL during March-April 2008. The depth of information that was obtained during the survey may not be representative of standard pharmacovigilance reporting as considered in this article, due to the short duration of the survey. Notably, the aim of the ALIVE feasibility study was primarily to assess the adherence to and acceptability of AL, and results showed that patients believed AL to be a good drug [15]. This could have biased their judgment such that they did not report minor AEs that might have occurred because this conflicted with their belief that the drug was excellent. Using passive reporting it has been suggested that reporting rates could be improved by providing non-financial incentives to community members and healthcare workers and ensuring confidentiality [30]. It has also been proposed that the lack of local expertise in pharmacovigilance could be tackled through developing exchange programmes with the major drug regulatory agencies and sharing of best practice, with the long-term goal that each country should establish its own national pharmacovigilance system that would contribute to a global database such as that held by the Uppsala Monitoring Centre [31].

The pharmacovigilance monitoring established for this study had the advantage of being a joint initiative between the investigators, the relevant drug regulatory authority and the sponsor (here, as in many instances, the drug manufacturer) - an approach which has recently been advocated [30,31]. This resulted in a valuable combination of local knowledge, utilization of an existing healthcare and technical infrastructure, regulatory skills, funding, and experience of safety monitoring and data management. Additionally, training and the provision of materials spanned all levels of healthcare personnel although the decline in AE reporting during the 18-month delay between initial training and follow-up training may indicate that more frequent repetition of training, with regular reminders, could have increased the number of events reported. Prompt training of new healthcare staff would also be beneficial. Lastly, use of SMS texts to notify the ALIVE safety database coordinator at the time an AE was identified ensured prompt data capture and guaranteed that the event would not be lost during its progress through the subsequent reporting channels.

In the ALIVE pharmacovigilance system reported here, it was observed that most AEs resolved in parallel with improvement in the treated clinical malaria. However, the relatively short half-life of artemether indicates that if an AE were related to the drug, drug clearance would have coincided with clearance of parasites (and host-response inflammatory markers) [32,33]. Lumefantrine, in contrast, has an extended half-life of 4-6 days [34]. Because this study included neither measurement of artemether or lumefantrine plasma concentration nor ascertainment of parasitaemia at recovery, it is likely that these AEs were related to malaria but the contribution of treatment cannot be excluded.

In conclusion, this article presents a practical model for pharmacovigilance monitoring during a prospective observational study that is applicable for rural community settings in sub-Saharan Africa or other developing regions. Training of healthcare workers at all levels to support protocol-mandated safety monitoring requirements was straightforward to undertake and showed a positive impact on the identification and safety reporting, but more frequent refresher courses and reminders are required to optimize and sustain reporting levels over time. Use of SMS texts is a pragmatic solution to communication challenges and helps to avoid lost or delayed reports. Finally, a collaborative approach involving all major participants - investigators, sponsor and regulatory authorities - from the outset offers a valuable template for future studies and facilitates sensitization of healthcare workers to the need for safety reporting. It is the authors' view that these strategies could support the achievement of safety monitoring requirements during an observational study. Moreover, they could contribute to improvements in ongoing spontaneous reporting of AEs to health authorities, marketing authorization holders and the WHO Collaborating Centre for International Drug Monitoring (the Uppsala Monitoring Centre) [35] in regions with limited experience of pharmacovigilance monitoring and in which local resources are restricted.

## Conflicts of interests

BG and AMK have received honoraria and travel grants from Novartis Pharma to present study findings at various international conferences. RS and NM are employees of Novartis. CM is an employee of the Tanzania Food and Drugs Authority. CL and AM have no conflicts of interests. A medical writer assisted with editing of a draft manuscript prepared by AMK.

## Authors' contributions

AMK contributed to study design, was a study investigator and drafted the manuscript for input by the other authors. NM acted as the safety reporting advisor to the project and facilitated the training of safety reporting staff. CM provided input and advice from the TFDA and acted as focal person for communication between the TFDA and the project. AM undertook data collection and facilitated the training of safety reporting staff. RS contributed to study design and provided input to the manuscript. CL participated in data interpretation and provided input to the manuscript. BG contributed to study design, facilitated the training of safety reporting staff and contributed to the writing of the manuscript.

All authors read and approved the final manuscript.

## Sources of funding

Funding was provided by Novartis Pharma AG, Basel, Switzerland, and the Novartis Foundation for Sustainable Development, Basel, Switzerland.
